# Advances in Tea Chemistry

**DOI:** 10.3390/foods12213944

**Published:** 2023-10-28

**Authors:** Qing-Qing Cao, Ying Gao, Yong-Quan Xu

**Affiliations:** Tea Research Institute, Chinese Academy of Agricultural Sciences, Key Laboratory of Biology, Genetics and Breeding of Special Economic Animals and Plants, Ministry of Agriculture and Rural Affairs, 9 South Meiling Road, Hangzhou 310008, China; caoqingqing@tricaas.com

The origins of tea, a traditional beverage in China, can be traced back to the Shennong period, about 2737 years before the birth of Christ [1]. Nowadays, with more than 2 billion cups consumed daily, tea is recognized as the most popular of the top three non-alcoholic beverages (i.e., tea, coffee, and cocoa), coming second only to water. The tea plant (*Camellia sinensis*) is now grown commercially in more than 60 countries, where it has made great contributions to local economies [2]. According to the statistical data from the UN Food and Agriculture Organization (FAO), the total global production of tea in 2020 was more than 7 million tons, with China, the largest producer, accounting for 42% of that figure [3].

Tea’s long history and widespread popularity can be attributed to its unique and gratifying flavor, along with numerous health benefits such as its anti-oxidant, anti-obesity, and anti-microbial properties [4]. An in-depth look reveals that tea contains abundant components with both flavor- and health-enhancing attributes, as well as the already well-known polyphenols, caffeine, and amino acids. These naturally occurring ingredients in tea plants undergo a series of complicated biochemical or physicochemical reactions during tea processing, converting them into new substances that work together to contribute to the final quality of tea-based beverages.

There are six categories of traditional tea in China, popularly known as green tea, yellow tea, white tea, oolong tea, black tea, and dark tea. Due to each one having its own specific manufacturing process, in which the degree of fermentation is a decisive factor, they are very different from one another in terms of their sensory appeal and physicochemical properties. For example, green tea, an unfermented tea variety, usually appears green in color both in terms of its leaves and the final infusion, as well as smelling refreshing and having an umami-dominated flavor. Meanwhile, black tea, with its reddish appearance and caramelized flavor, is made by applying full fermentation. Apart from the traditional teas mentioned above, since 2015 a range of “new tea drinks” have been setting off a burst of enthusiasm among young people around China, even becoming an emerging industry in their own right [5]. New tea drinks are an innovative field of beverage manufacture—with labels invoking fashion, health, and nature—taking traditional tea as their starting point and combining other food ingredients including, but are not limited to, milk, cream, fresh fruits, flowers, and so forth. The new tea industry is burgeoning, yet it has not received the attention it is due from researchers, encompassing topics such as how to successfully blend diverse materials so as to harmoniously shape divergent flavors and functions.

A plethora of studies have been conducted to explore associations between tea’s sensory qualities, particularly its flavor, with the chemicals that emerge during tea processing. We have so far detected about 600 volatile components in tea leaves and tea drinks, each of them with different scents, like grassy, woody, floral, etc. [6]. These aromatic compounds gather in different ratios for different teas, then combine to form characteristic aromas. Similarly, the taste of tea or its mouthfeel is induced by non-volatile extracted chemicals in the tea infusion—e.g., the catechins responsible for bitterness and astringency or the theanine that creates an umami taste [4]. The flavor of tea is determined by multiple factors, including aroma and taste or mouthfeel, but we know little about how these things work together. Indeed, the interaction between aroma and taste has been a research challenge even beyond tea.

The healthcare functions of tea come from its rich bioactive ingredients, especially flavonoids. The exploration of tea’s functional components has always attracted a great deal of research interest. The therapeutic potentials of quite a few of tea’s components have been confirmed repeatedly, and the mechanisms involved, such as EGCG, have also been deeply studied. At the same time, however, the limits of these potentials have also been realized; like the low stability and bioavailability of these components, which limit their utilizability. Moreover, figuring out how to extract, separate, and concentrate those bioactive components in an environmentally friendly, low-cost, and high-performance manner is also worthy of focus.

We launched this Special Issue of Foods, entitled “Advances in Tea Chemistry”, with the aim of publishing high-quality research on tea chemistry from a wide range of aspects, including but not limited to the sensory/flavor qualities of tea, tea processing and storage, the extraction, bioactivity, and utilization of functional components from tea, as well as new tea-based beverages or foods. This series will be useful for expanding our understanding of the associations between tea chemistry, flavor, and bioactivity.

## References

[1] Wambulwa M.C., Meegahakumbura M.K., Kamunya S., Wachira F.N. (2021). From the wild to the cup: Tracking footprints of the tea species in time and space. Front. Nutr..

[2] Drew L. (2019). Making tea. Nature.

[3] Zhai X., Zhang L., Granvogl M., Ho C.-T., Wan X. (2022). Flavor of tea (Camellia sinensis): A review on odorants and analytical techniques. Compr. Rev. Food Sci. Food Saf..

[4] Zhang L., Cao Q.-Q., Granato D., Xu Y.-Q., Ho C.-T. (2020). Association between chemistry and taste of tea: A review. Trends Food Sci. Technol..

[5] Lu D. Analyze the Marketing Strategies of New-tea Drinks Industry by the SWOT and PEST Tools-Take Nayuki as an Example. Proceedings of the 2022 7th International Conference on Social Sciences and Economic Development (ICSSED 2022).

[6] Zhou Y., He Y., Zhu Z. (2023). Understanding of formation and change of chiral aroma compounds from tea leaf to tea cup provides essential information for tea quality improvement. Food Res. Int..

